# Brain Tumor Segmentation Using U‐Net With ResNet50 Encoder for Enhanced MRI Analysis

**DOI:** 10.1155/ijbi/5318118

**Published:** 2026-06-10

**Authors:** Abeer Masfer Alkahtani, Samia Dardouri

**Affiliations:** ^1^ Department of Computer Science, College of Computing and Information Technology, Shaqra University, Shaqra, Saudi Arabia, su.edu.sa; ^2^ Innov′COM Laboratory-Sup′Com, University of Carthage, Tunis, Tunisia, ucar.rnu.tn

**Keywords:** brain tumor, deep learning (DL), magnetic resonance imaging (MRI), ResNet50, segmentation, U-Net

## Abstract

Accurate brain tumor segmentation in magnetic resonance imaging (MRI) remains a challenging task due to the high variability in tumor appearance, shape, and location. Manual segmentation is time‐consuming, subjective, and impractical for large‐scale clinical use, highlighting the need for robust automated solutions. This study introduces an enhanced U‐Net architecture with a ResNet50 encoder, designed to improve feature extraction through deeper convolutional layers and residual connections. By reformulating tumor delineation as a pixel‐level segmentation problem rather than image‐level classification, the model achieves more precise boundary detection. Trained on the publicly available TCGA‐LGG dataset, the proposed model significantly outperformed the baseline U‐Net, achieving a Dice score of 0.9659, an Intersection over Union (IoU) of 0.9567, and a Matthews correlation coefficient (MCC) of 0.9253. These results demonstrate superior segmentation capability compared to standard U‐Net and are competitive with recent state‐of‐the‐art methods. The findings highlight the potential of the proposed framework as a proof of concept for integration into clinical decision support, while also underscoring the need for future validation on larger, multi‐institutional datasets.

## 1. Introduction

The brain is a central element of the mortal nervous system, responsible for coordinating a wide range of physiological and cognitive processes. Uncontrolled cell growth within brain tissues can lead to tumor formation, which may significantly impair neurological function and threaten patient survival. According to global cancer statistics, more than 300,000 new cases of primary brain and central nervous system (CNS) tumors were reported worldwide in 2020, highlighting the growing clinical burden of this disease [1]. In Saudi Arabia, epidemiological analyses based on public cancer registry data have furthermore demonstrated variations in tumor prevalence across regions and demographic groups, emphasizing the need for effective diagnostic strategies.

Magnetic resonance imaging (MRI) is extensively regarded as the most effective imaging modality for brain tumor assessment due to its high‐resolution visualization of soft tissues. Multimodal MRI sequences, including T1, T1‐laden discrepancy‐enhanced (T1c), T2, and FLAIR, provide complementary information that is essential for accurate tumor identification and characterization. Still, precise delineation of tumor regions from MRI scans remains a challenging and labor‐intensive task. Manual segmentation performed by radiologists is time‐consuming, subject to interobserver variability, and difficult to scale for large datasets, thereby limiting its practicality in routine clinical workflows.

To address these challenges, computer‐aided diagnosis (CAD) systems have been developed to help clinicians in detecting and localizing brain tumors with reduced human intervention [2]. The integration of machine learning and deep learning techniques into CAD systems has significantly improved diagnostic performance, enabling more accurate classification, segmentation, and analysis of medical images [3]. In particular, deep learning has converted medical image segmentation by allowing models to learn hierarchical and spatial features directly from raw data.

Among the various deep learning architectures, the U‐Net model has become a standard approach for biomedical image segmentation due to its encoder–decoder structure and skip connections, which facilitate the preservation of fine‐grained spatial information [4]. Despite its strong performance, the conventional U‐Net architecture may struggle to capture complex tumor characteristics, especially in cases involving high variability in tumor morphology and imaging conditions.

Brain tumors exhibit considerable diversity in terms of shape, size, texture, and intensity, making it difficult for a single model to generalize across different case data. Also, MRI datasets frequently suffer from inconsistencies such as noise, variations in accession protocols, and deficient or low‐quality reviews. These factors further complicate the segmentation process and can negatively impact model performance.

Recent studies have explored hybrid architectures that integrate U‐Net with pretrained convolutional neural networks to enhance feature extraction capabilities. In particular, residual networks such as ResNet50 have demonstrated effectiveness in learning deep hierarchical representations while mitigating the vanishing gradient problem through residual connections. Such architectures enable improved training stability and more effectively capture of complex image features, which are essential for accurate tumor segmentation.

Likewise, although deep learning models have shown promising results in exploration surroundings, there remains a gap between experimental performance and real‐world clinical connection. Accurate tumor segmentation plays a pivotal role in diagnosis, treatment planning, and disease monitoring, as it enables clinicians to accurately identify tumor boundaries and assess progression over time. Thus, developing robust, effective, and reproducible segmentation models is of critical significance.

In this environment, the present study proposes an enhanced U‐Net architecture incorporating a ResNet50 encoder for improved brain tumor segmentation from MRI scans. The proposed model is specifically optimized for the TCGA‐LGG dataset, with careful design of the encoder–decoder structure and feature extraction process. To ensure a fair evaluation, a controlled benchmarking framework is adopted, where the proposed model is compared with a baseline U‐Net under identical experimental conditions.

In addition, this study investigates the impact of different loss functions, including binary cross‐entropy (BCE), focal loss, and Jaccard loss, selecting the most effective approach based on empirical performance. Alongside commonly used evaluation metrics such as accuracy, the Dice score, and Intersection over Union (IoU), the Matthews correlation coefficient (MCC) is incorporated to provide a balanced evaluation, particularly in the presence of class imbalance.

Overall, this work is aimed at developing a computationally effective and high‐performing segmentation framework that addresses crucial challenges in brain tumor analysis while maintaining potential for future clinical integration.

## 2. Related Works

Brain tumor segmentation from MRI represents a fundamental task in modern medical image analysis because of its direct impact on clinical diagnosis, treatment planning, and patient prognosis. The increasing prevalence and mortality associated with brain and CNS tumors, as stressed in recent global cancer statistics [1, 5], emphasize the growing demand for accurate, automated diagnostic technologies capable of aiding clinicians in routine practice.

Before tumor segmentation approaches primarily reckoned on handcrafted image features and conventional machine learning algorithms. Although these methods contributed to initial progress in automated analysis, their effectiveness was frequently constrained by sensitivity to imaging noise, variability in tumor morphology, and the need for manual feature extraction. The emergence of deep learning significantly transformed this field by enabling neural networks to automatically learn hierarchical representations directly from imaging data, thereby improving segmentation capability and robustness.

Among deep learning–based segmentation frameworks, the U‐Net architecture proposed by Ronneberger et al. has become one of the most influential models in biomedical image segmentation. Its encoder–decoder structure, combined with skip connections, enables effective preservation of both contextual and spatial information, making it highly suitable for dense pixel‐level prediction tasks. However, despite its widespread adoption and strong performance, the conventional U‐Net architecture may face difficulties in accurately segmenting highly heterogeneous tumor structures and complex imaging patterns.

To address these limitations, recent studies have explored multitudinous architectural advancements and hybrid segmentation strategies aimed at enhancing feature extraction and segmentation accuracy. Several approaches have integrated multiscale learning and attention mechanisms to improve boundary localization and contextual understanding. For instance, previous studies involving multiscale attention‐based U‐Net models combined with EfficientNet encoders demonstrated enhanced delineation of tumor boundaries and improved segmentation sensitivity [2]. Other studies have proposed lightweight U‐Net variants optimized for computational efficiency and real‐time clinical applications while maintaining competitive segmentation performance [3]. Bayesian learning extensions have also been investigated to improve model interpretability alongside segmentation accuracy [6].

Recent developments have concentrated on improving edge preservation and contextual feature representation. Edge‐aware segmentation architectures, such as EdgeU‐Net, were introduced to enhance tumor boundary detection and improve localization accuracy [7]. Contextual feature fusion strategies and redesigned skip connections have been employed to strengthen segmentation performance on standard datasets like BraTS [8]. Likewise, lightweight three‐dimensional segmentation frameworks incorporating parallel attention mechanisms have demonstrated promising trade‐offs between computational complexity and segmentation accuracy, making them suitable for practical clinical environments [9].

More recently, hybrid architectures combining convolutional neural networks with transformer‐based attention mechanisms have gained increasing interest in medical image segmentation research. Transformer‐integrated frameworks, including TransUNet proposed by Chen et al. [10], use self‐attention mechanisms to capture long‐range dependencies within medical images. Although these approaches improve global contextual understanding, they frequently require substantial computational resources. In contrast, CNN‐based hybrid frameworks integrating EfficientNet encoders with U‐Net architectures have achieved strong performance in multiclass tumor segmentation tasks while maintaining computational efficiency [11]. Other studies have employed nested dilated convolution strategies to enlarge receptive fields and improve tumor boundary localization performance [12].

Table 1 summarizes representative recent approaches for brain tumor segmentation, highlighting variations in datasets, architectural designs, evaluation criteria, and reported limitations. Taken together, these studies demonstrate the rapid evolution of segmentation methodologies from traditional machine learning techniques toward advanced deep learning–based frameworks capable of learning increasingly complex image representations.

**Table 1:** Overview of the brain tumor dataset used for segmentation.

Ref	Dataset	Method	Accuracy	
6	BraTS 2020	U‐Net + Bayesian machine learning (explainable AI)	97.75%	Computationally intensive; requires expert interpretation
7	Clinical MRI (unspecified)	Edge‐aware U‐Net variant	97.6%	Sensitive to image noise and edge variation
10	Synapse (MRI, general use)	TransUNet (transformer + U‐Net encoder)	77.48%	Requires high memory and long training time due to transformers
12	MRI brain scans	Nested dilation convolutional networks	Dice: 0.91; IoU: 0.85	High memory usage; performance varies with tumor shapes
15	Brain MRI scan	SVM (support vector machine)	Accuracy: 92.6%	Performance may depend on the quality and characteristics of the MRI scans
16	Brain MRI scan	CNN‐based tumor detection	Accuracy: 94.7%	Needs labeled data; affected by tumor diversity and imbalance
19	MRI scans	EfficientNetB7 + U‐Net (MRI slice optimization)	Dice: ~0.86; IoU: ~0.78 (similar studies)	High computational demand; requires extensive training data
21	TCGA histopathology (MRI‐linked)	Optimized CNN (with batch size, augmentation)	Not specified (performance improved with tuning)	Needs better data labeling; limited impact from augmentation strategies
Proposed	TCGA‐LGG (brain MRI)	U‐Net + ResNet50 encoder	Accuracy: 96.61%; Dice: 0.9659; MCC: 0.9253	Outperforms baseline U‐Net on the same dataset

Beyond purely deep learning approaches, several examinations have explored hybrid systems that combine deep feature extraction with classical machine learning methods. For instance, some researchers employed pretrained convolutional neural networks together with conventional classifiers to improve tumor classification accuracy [13]. Prior work also incorporated image processing techniques alongside machine learning algorithms such as support vector machines (SVMs) and fuzzy conclusion systems for tumor analysis [14, 15]. Ensemble learning strategies have furthermore been introduced to improve robustness and generalization across diverse datasets [16]. Modified U‐Net architectures, including BU‐Net and other enhanced variants, have also demonstrated improved segmentation performance in automated MRI analysis systems [17–37].

Overall, the evolution of brain tumor segmentation methods reflects a transition from traditional handcrafted approaches to advanced deep learning–based frameworks. Enhancements such as attention mechanisms, multiscale feature extraction, and hybrid encoder–decoder architectures have consistently contributed to improved segmentation accuracy and robustness.

Building on these advancements, the present study proposes a U‐Net architecture enhanced with a ResNet50 encoder. By leveraging residual learning, the proposed model improves feature extraction, facilitates deeper network training, and enhances gradient propagation. This approach is aimed at achieving high segmentation accuracy while maintaining computational efficiency, addressing key challenges associated with real‐world clinical deployment.

## 3. Materials and Methods

### 3.1. Description of Brain MRI Segmentation Dataset for Lower Grade Glioma (TCGA‐LGG)

The dataset employed in this study is the brain MRI segmentation dataset for lower grade glioma (TCGA‐LGG), publicly released by Buda on Kaggle [38]. It contains multimodal MRI scans with manually annotated FLAIR abnormality segmentation masks. The data are sourced from The Cancer Imaging Archive (TCIA) and correspond to 110 patients, with each patient folder including multiple 2D MRI slices and their corresponding segmentation masks in TIFF format, totaling more than 7860 slices.

While this dataset provides high‐quality annotations and has been widely adopted in research, its limited cohort size (110 patients) and reliance on 2D slices rather than full 3D volumetric scans may restrict the model′s generalizability across broader clinical populations. We therefore consider this study as a proof of concept, and future work will aim to validate the model on larger, multi‐institutional, and 3D MRI datasets.

**Figure 1:**
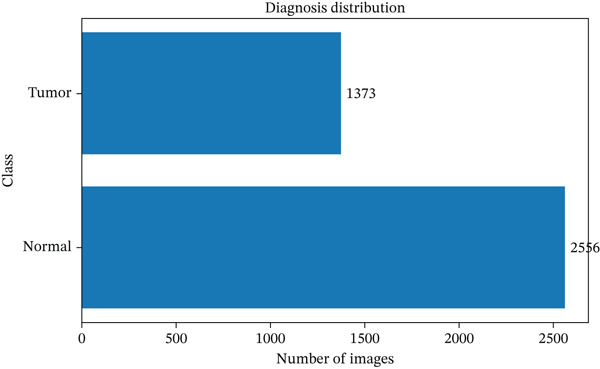
Distribution of the dataset.

To address the issue of imbalanced data in brain tumor classification, as shown in Figure 1, data augmentation techniques were employed to artificially expand the dataset and create a more balanced distribution of tumor images. Various augmentation methods, including rotation, flipping, scaling, contrast adjustments, and elastic deformations, were applied to enhance the diversity of the tumor images while preserving their essential features. These transformations improve the model′s generalization by exposing it to a wider range of variations, reducing overfitting, and boosting performance in real‐world clinical environments. Through data augmentation, the dataset becomes more evenly distributed, enabling the deep learning model to learn more effectively and increase accuracy in tumor detection and classification. Table 2 presents the class distribution of the TCGA‐LGG dataset before and after the application of data augmentation techniques.

**Table 2:** Class distribution before and after data augmentation.

Class	Original images	After augmentation
Normal	2556	2556
Tumor	1373	2556
Total	3929	5112

### 3.2. Data Preprocessing

Preprocessing included path extraction using the glob module, construction of a Pandas DataFrame to map each image with its mask and patient ID, and dataset splitting into training (70%), validation (20%), and testing (10%) using stratified sampling to preserve class distribution. Image processing involved resizing slices to 256 × 256 or 320 × 256 pixels, applying random brightness/contrast changes and horizontal and vertical flips using augmentations, normalizing pixel values to the [0, 1] range, and converting images to PyTorch tensors. PyTorch′s DataLoader was used for efficient minibatch loading during training.

### 3.3. Model Architecture

**Figure 2:**
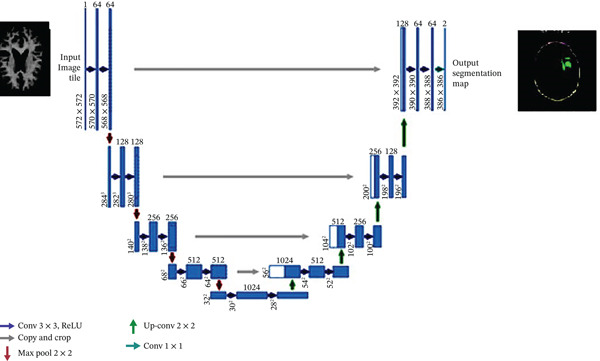
Architecture of a U‐Net model for brain tumor segmentation.

The proposed model is a modified U‐Net architecture (Figure 2) enhanced with a ResNet50 encoder for accurate brain tumor segmentation in MRI scans. Widely adopted in medical image segmentation, the U‐Net is adapted here by increasing the convolutional depth up to 1024 filters and configuring block sizes as 64, 128, 256, 512, and 1024 to better capture complex tumor structures. The encoder, based on ResNet50 pretrained on ImageNet, extracts rich hierarchical features through deep residual learning, while the decoder path performs progressive upsampling using 2 × 2 transposed convolutions, concatenated with corresponding encoder outputs via skip connections to preserve spatial detail. Repeated 3 × 3 convolutions with ReLU activation and 2 × 2 max pooling are used throughout the contracting path, and refinement is performed in the expansive path. During preliminary experiments, we evaluated BCE and focal loss to address class imbalance and improve boundary segmentation. However, for the final model training and evaluation, we selected Jaccard loss as the loss function, since it demonstrated superior convergence behavior and segmentation accuracy compared to BCE and focal loss. The final 1 × 1 convolution with sigmoid activation outputs a high‐resolution binary segmentation map, enabling precise localization of brain tumors.

**Figure 3:**
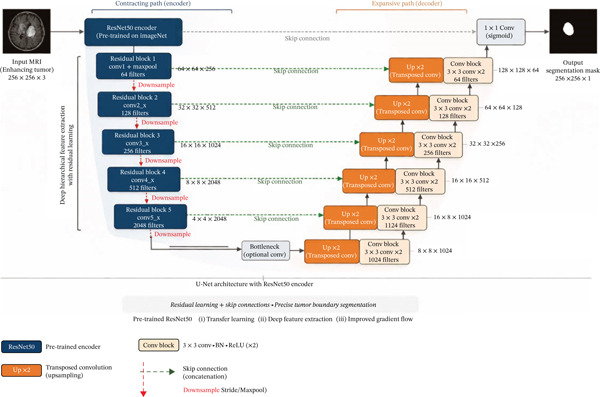
Proposed model.

Figure 3 illustrates the architecture of the proposed model, a modified U‐Net enhanced with a ResNet50 encoder for brain tumor segmentation in MRI scans. The diagram highlights the deep contracting path utilizing residual blocks from ResNet50, the symmetrical expansive path for upsampling and refinement, and the skip connections that enable precise spatial reconstruction of tumor boundaries. Key components such as the increasing convolutional depth, transposed convolutions, and the final 1 × 1 output layer are visually represented to convey the full segmentation pipeline. ResNet50 is employed as the encoder in the proposed architecture to extract deep and hierarchical features from the input medical images. Its residual learning framework, consisting of 50 layers, enables efficient training of deep networks by mitigating the vanishing gradient problem through shortcut connections. These residual blocks facilitate better feature propagation and faster convergence, making ResNet50 highly effective for complex tasks like brain tumor segmentation. The encoder leverages convolutional layers, batch normalization, and ReLU activation to progressively capture spatial and semantic details. By using a pretrained ResNet50 on large‐scale datasets, the model benefits from transfer learning, enhancing generalization and reducing the need for extensive labeled medical data.

**Figure 4:**
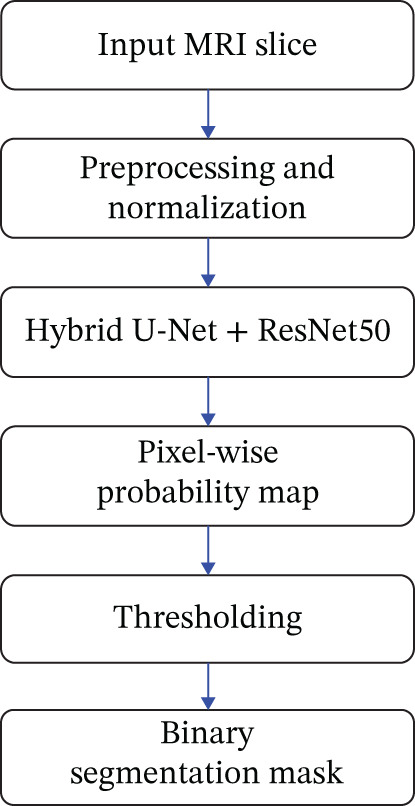
Flowchart or diagram of segmentation process.

The overall segmentation workflow illustrated in Figure 4 begins with preprocessing and normalization of the input MRI slices to ensure consistent intensity distributions and reduce variability across scans. These preprocessed slices are then fed into the proposed hybrid U‐Net with a ResNet50 encoder, which generates a pixel‐wise probability map indicating the likelihood of tumor presence. To obtain the final prediction, the probability map is thresholded, producing a binary segmentation mask that delineates tumor regions from healthy tissue. Finally, the resulting masks are quantitatively evaluated against the ground truth annotations using the selected performance metrics, thereby assessing the accuracy and robustness of the proposed approach.

## 4. Results

### 4.1. Implementation Details

The proposed brain tumor segmentation model was implemented using Python 3.8.10 and PyTorch 1.11.0. All experiments were conducted on a high‐performance server equipped with an AMD EPYC 7551P processor (AMD, Santa Clara, California, United States) and a single NVIDIA RTX A5000 GPU (24 GB VRAM; PNY, Parsippany, New Jersey, United States). The model utilized an initial learning rate of 3.00 × 10^−4^, and due to memory limitations, the batch size was set to 32. To ensure efficient convergence and training stability, the Adam optimizer was employed. The key configuration parameters of the training process are summarized in Table 3.

**Table 3:** Model parameter configuration.

Basic configuration	Value
PyTorch version	1.11.0
Python	3.8.10
GPU	NVIDIA RTX A5000 (24 GB)
Learning rate	0.0001
Optimizer	Adam
Batch size	32
Epoch	100
Input size	128 × 128 × 128
Output size	128 × 128 × 128
Random seed	Jaccard loss
Loss function	42
Learning rate scheduler	Cosine annealing

### 4.2. Evaluation Metrics

The analysis of the experiment was performed according to the following performance parameters.

#### 4.2.1. Accuracy

Accuracy represents the proportion of correctly identified pixels in a brain tumor image, including both tumor and nontumor regions, as illustrated in Equation (1) [39, 40]:

(1) Accuracy=TP+TNTP+TN+FP+FN.

#### 4.2.2. Precision

Precision measures the accuracy of positive brain tumor detections by quantifying how many of the predicted tumor regions correctly match the ground truth [39], as detailed in Equation (2):

(2) Precision=TPTP+FP.

#### 4.2.3. Sensitivity

Sensitivity, also called true positive rate, is the percentage of true positive values that are accurately detected, as detailed in Equation (3):

(3) Sensitivity=TPTP+TN.

#### 4.2.4. Specificity

Compared to sensitivity, specificity or the true negative rate is a percentage of correctly detected negative values [41], and it is expressed in Equation (4):

(4) Specificity=TNFP+TN.

#### 4.2.5. Dice Score

The Dice score (also known as the Dice similarity coefficient or DSC) is another measure of similarity between two sets. The Dice coefficient is a widely used and intuitive metric that quantifies the overlap between predicted and ground truth regions, making interpretation straightforward. It is particularly effective for imbalanced datasets, as it equally penalizes false positives and false negatives, ensuring balanced evaluation. This makes it especially valuable in biomedical imaging, where detecting small or subtle structures is critical. The Dice coefficient is suitable for both binary and multiclass segmentation tasks, contributing to its popularity in medical image analysis and other segmentation‐focused applications [41]. The Dice score is defined in Equation (5):

(5) Dice score=2∗TP2∗TP+FN+TN.

#### 4.2.6. Jaccard Index

The Jaccard index (also known as the Jaccard similarity coefficient) is a measure of similarity between two sets. It is defined as the size of the intersection of the sets divided by the size of the union of the sets. It is a measure of similarity between two datasets that is straightforward to compute but can be easily misinterpreted [41]. It is expressed in Equation (6):

(6) Jaccard index=TPTP+FN+TN.

#### 4.2.7. MCC

The MCC is a performance metric that evaluates the correlation between actual and predicted classifications. It considers all four components of the confusion matrix: true positives, true negatives, false positives, and false negatives, providing a balanced measure even in the presence of class imbalance. The MCC is calculated using Equation (7):

(7) MCC=TP∗TN−FP∗FNTP+FPTP+FNTN+FPTN+FN.

## 5. Discussion

Despite the strong segmentation performance shown by the proposed model, there is an important limitation related to the dataset. The TCGA‐LGG dataset only includes 110 patients and is based on 2D MRI slices, which might not fully capture the spatial context needed for complex tumor shapes. This small sample size could limit how well the model works with different groups of patients in real‐world settings. Future research should aim to test the model on larger, multicenter datasets and use 3D MRI scans to improve its reliability and practical use. While some studies report higher accuracy, many of these results come from different datasets, often with more patients, different types of imaging, or different tasks like classification instead of pixel‐level segmentation. Within the TCGA‐LGG dataset used in this study, the U‐Net model using a ResNet50 encoder outperforms the standard U‐Net, showing its effectiveness for binary tumor segmentation.

**Figure 5:**
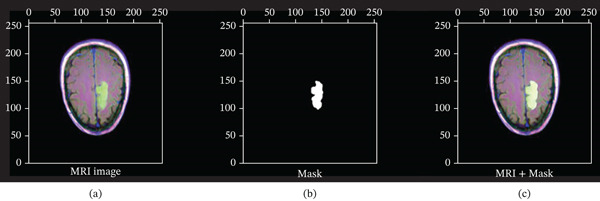
(a–c) Segmentation results.

For comparison, we trained a standard U‐Net model under the same conditions on the same dataset. The standard model achieved an accuracy of 0.9432, a Dice score of 0.9325, and an MCC of 0.8810. In comparison, the proposed model with the ResNet50 encoder improved performance on all these metrics, achieving an accuracy of 0.9661, a Dice score of 0.9659, and an MCC of 0.9253. This comparison shows the benefit of using the ResNet50 encoder in the U‐Net architecture for brain tumor segmentation. Figure 5 shows three panels that illustrate the segmentation results of a U‐Net model on a brain MRI scan. Figure 5a shows the original MRI scan, where a bright area suggests the presence of a tumor. Figure 5b shows the corresponding segmentation mask, where the tumor area is clearly marked in white, showing that the model successfully detects it. Figure 5c overlays the mask on the MRI scan, effectively showing the tumor area in green. These results suggest that the model accurately identifies and segments the tumor area, supporting its effectiveness in detecting abnormalities in medical imaging. We note that while BCE and focal loss were initially tested, the final model performance reported in this study is based solely on Jaccard loss. Future work could explore combining different loss functions, such as Jaccard and Dice loss, to further improve segmentation performance. As shown in Table 4, Jaccard loss achieved the best segmentation performance across all evaluation metrics, including Dice (0.9659), IoU (0.9567), accuracy (0.9661), and MCC (0.9253). Although focal loss improved performance compared to BCE, Jaccard loss showed better overlap optimization and more consistent convergence, especially when defining tumor boundaries. Therefore, Jaccard loss was chosen as the final loss function for training the proposed model.

**Table 4:** Comparative performance of loss functions.

Loss function	Dice score	IoU	Accuracy	MCC
Binary cross‐entropy (BCE)	0.9454	0.9331	0.9554	0.8945
Focal loss	0.9545	0.9456	0.9652	0.9134
Jaccard loss (selected)	0.9659	0.9567	0.9661	0.9253

**Figure 6:**
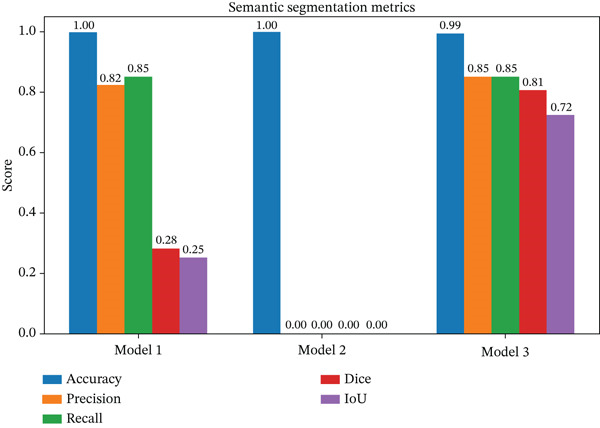
Performance metrics including accuracy, precision, recall, Dice score, and IoU.

The bar chart in Figure 6 presents the semantic segmentation performance of the U‐Net model using key metrics, including accuracy, precision, recall, Dice score, and IoU. The model achieves near‐perfect accuracy (close to 1.0) across different categories, demonstrating strong overall performance. However, variations in precision and recall indicate potential challenges in balancing false positives and false negatives when identifying tumor regions. The Dice score and IoU, which are critical for assessing segmentation quality, are comparatively lower in one category, suggesting areas for improvement in accurately delineating tumor boundaries. Despite these minor limitations, the model exhibits reliable segmentation capabilities, making it effective for medical image analysis.

**Figure 7:**
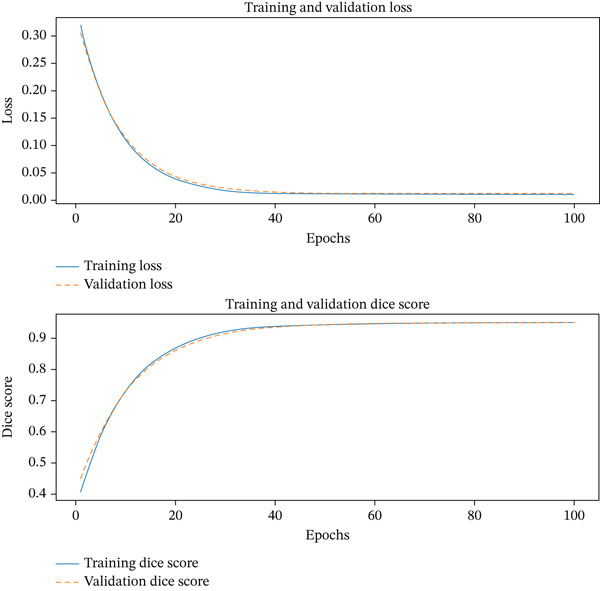
Training and validation loss curves along with Dice score trends.

The training curves in Figure 7 indicate a well‐optimized segmentation model with effective learning and minimal overfitting. The loss curve steadily decreases and stabilizes near zero, while the Dice score, a key segmentation metric, rapidly improves before leveling off around 0.9, suggesting high segmentation accuracy. The close alignment between training and evaluation metrics confirms strong generalization, ensuring reliable tumor segmentation. These results highlight the model′s robustness, though further fine‐tuning, such as data augmentation or regularization, could further enhance performance.

The U‐Net model with a ResNet50 encoder proposed in this study exhibited robust segmentation performance, achieving a Dice score of 0.9659, an accuracy of 0.9661, and an MCC of 0.9253. These results represent a significant improvement over the baseline U‐Net, particularly in accurately delineating tumor boundaries, highlighting the model′s enhanced precision and effectiveness in brain tumor segmentation.

**Figure 8:**
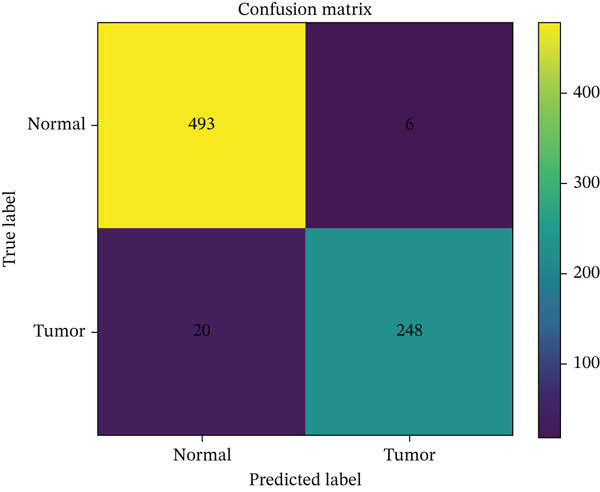
Confusion matrix of the proposed model.

The confusion matrix, illustrated in Figure 8, showcases the performance of the U‐Net model with a ResNet50 encoder in classifying normal and tumor cases from MRI scans. The model correctly predicted 493 normal and 248 tumor cases, with only 6 false positives and 20 false negatives. These results highlight the model′s reliability in accurately distinguishing between normal and tumor regions, demonstrating its high accuracy and effectiveness in tumor detection.

Table 5 presents a focused comparison between the proposed U‐Net with ResNet50 encoder and several recent state‐of‐the‐art brain tumor segmentation models evaluated on TCGA‐LGG or closely related public MRI datasets. The comparison highlights commonly reported overlap‐based performance metrics, including Dice score, IoU, and, when available, MCC.

**Table 5:** Comparison with recent brain tumor segmentation models.

Study/model	Dataset	Architecture	Dice	IoU	MCC
Nested dilation network (Wang et al.)	MRI brain scans	Dilated CNN	0.91	0.85	—
EfficientNetB7 + U‐Net	MRI brain dataset	Hybrid encoder	~0.86	~0.78	—
TransUNet	Synapse MRI	Transformer + U‐Net	0.77	—	—
BU‐Net	MRI brain scans	Modified U‐Net	0.94	—	—
Baseline U‐Net	TCGA‐LGG	Standard U‐Net	0.9325	0.9184 ^∗^	0.8810
Proposed model	TCGA‐LGG	U‐Net + ResNet50	0.9659	0.9567	0.9253

*Note:* The asterisk means the IoU value taken from the corresponding reference.

As shown in the table, the proposed model achieves a Dice score of 0.9659 and an IoU of 0.9567, demonstrating strong overlap accuracy compared to recent hybrid and modified U‐Net architectures. Furthermore, the inclusion of MCC (0.9253) provides a balanced assessment of segmentation performance under class imbalance, a metric not consistently reported in comparable studies. While direct numerical comparison must be interpreted cautiously due to differences in datasets, preprocessing strategies, and evaluation protocols, the results indicate that the proposed approach is competitive with recent state‐of‐the‐art segmentation methods.

The proposed U‐Net framework with a ResNet50 encoder showed promising segmentation results, but this study should be considered mainly as a preliminary proof‐of‐concept exploration. The presented approach has not been evaluated in real clinical settings, so its practical effectiveness in normal radiologic workflows is unknown. A thorough clinical evaluation by experienced radiologists is needed to evaluate the reliability, interpretability, and potential for real diagnostic assistance of the system. Moreover, future work should include heterogeneous datasets from different medical centers and different imaging modalities to improve model robustness and generalization across clinical settings.

Another limitation of the current framework is the focus on binary segmentation, where the model only distinguishes between tumor and nontumor tissue sections. This is a major step forward for automated tumor delineation, but does not address the clinically relevant differentiation among tumor types such as gliomas, meningiomas, and pituitary tumors. Extending the approach to multiclass segmentation would be a great contribution to clinical utility as it would enable more thorough tumor characterization, diagnosis, and treatment planning.

## 6. Conclusion

In this work, we presented the construction and optimization of a deep learning framework for brain tumor segmentation by using an augmented U‐Net model with a ResNet50 encoder. Extensive experimentation with different training settings and optimization techniques has shown the proposed approach to be robust with many evaluation metrics such as accuracy, recall, F1 score, and MCC. The results underline the importance of optimization of the architecture and improvement of training in order to increase the reliability of automated medical image segmentation systems.

The proposed approach showed better segmentation performance than the traditional approaches with a consistent improvement in Dice coefficient, accuracy, IoU, and mean IoU. The stability observed during the training phase showed the robustness and reproducibility of the framework over many experimental conditions. The results show that the proposed model can be used as a powerful computational tool to assist in brain tumor investigation, diagnostics, and treatment planning applications.

Despite the encouraging results, there are still many constraints. The current evaluation was made on a small dataset of 2D MRI slices from a sample of 110 patients, which might limit the model′s generalizability to wider clinical scenarios. Furthermore, the system has not been validated in real radiology processes, so clinical assessment is a crucial next step before practical implementation. Future work will focus on validating the framework using larger and more heterogeneous data from multiple institutions, including volumetric 3D MRI acquisitions and other imaging modalities such as CT scans.

Additional development will investigate more complex segmentation architectures such as attention‐based models and hybrid deep learning approaches to improve the delineation of complex and irregular tumor borders. Combining data augmentation, semisupervised learning, and domain adaptation strategies may improve model generalization across different imaging environments and scanning protocols. In addition, incorporating explainable artificial intelligence methods, such as Grad‐CAM visualization, can improve interpretability and increase clinician confidence in AI‐driven diagnostic tools.

This work will be further extended to explore multiclass tumor segmentation to differentiate between tumor subtypes such as gliomas, meningioma, and pituitary tumors. These improvements will enhance the therapeutic value of the framework, allowing tumor grading and classification together with segmentation. Future study will include radiologist‐in‐the‐loop validation and pilot clinical deployment studies to assess usability, efficiency, and real‐world effectiveness in hospital settings. Together, these directives are aimed at establishing the proposed framework as a robust and clinically applicable solution for the diagnosis and treatment of brain tumors.

## Author Contributions

Abeer Masfer Alkahtani: writing—original draft, coding; Samia Dardouri: writing—review and editing, validation.

## Funding

No funding was received for this manuscript.

## Conflicts of Interest

The authors declare no conflicts of interest.

## Data Availability

The data are openly available in a public repository (https://www.kaggle.com/datasets/mateuszbuda/lgg-mri-segmentation/data).
